# Vincent's Angina

**Published:** 1909-05-01

**Authors:** 


### VINCENT'S ANGINA.

Vincent's angina is a variety of tonsillitis or sore throat which is apt to be mistaken for diphtheria, especially as it may occur in epidemics.

Clinically the disease is at first membranous, and later ulcerous or ulcero-membranous, so that it is particularly at its commencement that it simulates diphtheria. After the second or third day ulceration occurs beneath the false membrane, and presently an undoubted ulcer is apparent. The membrane is fairly soft, whitish in colour, and gelatinous. It may occur not only upon the tonsils, but also upon the anterior and posterior pillars of the fauces, the uvula, the posterior pharyngeal wall, and the buccal mucosa.

The breath becomes foul; dysphagia is a more or less prominent symptom; and the voice is often husky, nasal, or otherwise abnormal. The submaxillary glands become enlarged. The temperature is most raised during the first day or two of the illness, generally reaching to something between 100° F. and 102.5° F. There is little or no tendency for the enlarged glands to suppurate. Neither skin rash nor articular symptoms occur.

The lesions are dependent upon a particular bacillus which is remarkably fusiform, elongated, and often somewhat curved. This micro-organism, which has been styled the *bacillus fusiformis*, retains the stain by Gram's method imperfectly. It occurs in enormous numbers upon the affected regions at the beginning of the illness, being often associated with streptococci, and notably with a peculiar spirillum. The detection of long fusiform bacilli and of these spirilla in stained films made from swabbings from the throat affords a positive diagnosis. The micro-organisms bear no resemblance to diphtheria bacilli.

The importance of distinguishing Vincent's angina from diphtheria is at least threefold. In the first place, the sending of a case into a diphtheria isolation ward would be a very grave thing, for the concurrence of the two diseases would be serious. In the second place, the administration of anti-diphtheritic serum in a case of Vincent's angina would have no specific effect. In the third place, the prognosis of Vincent's angina is much more favourable than is that of diphtheria.

*Treatment* consists of local application of antiseptic washes—chlorine water administered with a syringe, for example, or carbolic lotion, chinosol lotion, or formalin solution painted on with a soft brush or sprayed on with a suitable apparatus. In mild cases a simple gargle may be all that is required. Treated with care the disease ordinarily gets well in one, two, or three weeks, and there seems to be no danger of the serious sequelæ of diphtheria—heart failure or palatal paralysis or peripheral neuritis.

If, on the other hand, the condition is neglected, it may last a long time. Vincent himself, for instance, records the case of a man aged twenty-four who was ill with it for six months and more, with alternate periods of improvement and relapse. The left tonsil was at first the site of a false membrane which covered in a small ulcer, whilst at the same time there was a feeling of lassitude and illness, with loss of appetite, headache, pyrexia, up to 102° F., and inflammatory enlargement of the submaxillary lymphatic glands. During later exacerbations both tonsils were very red and swollen. Upon one or other there would be soft, greyish white, well demarcated false membrane, perhaps the area of a threepenny piece or rather more, covering a shallow ulcer. This condition would be accompanied by fœtor of the breath, difficulty in and pain on swallowing, severe headache, and pyrexia, without skin-rash and without albuminuria. Cultivations from swabbings of the throat revealed no Klebs-Lœffler bacilli. Films prepared from the false membrane contained multitudes of fusiform bacilli, associated with some spirilla and many micro-cocci. Local treatment carried out with greater assiduity than before led to a cure within a fortnight.

